# GATA3 maintains the quiescent state of cochlear supporting cells by regulating p27^kip1^

**DOI:** 10.1038/s41598-021-95427-3

**Published:** 2021-08-04

**Authors:** Jiadong Xu, Dongliang Yu, Xuhui Dong, Xiaoling Xie, Mei Xu, Luming Guo, Liang Huang, Qi Tang, Lin Gan

**Affiliations:** 1 College of Life Sciences, Zhejiang University, Hangzhou, 310058 Zhejiang China; 2 Department of Ophthalmology and Flaum Eye Institute, University of Rochester, Rochester, NY 14642 USA; 3 College of Life Sciences and Medicine, Zhejiang Sci-Tech University, Hangzhou, 310018 Zhejiang China; 4 Department of Otolaryngology, Peking Union Medical College Hospital, Chinese Academy of Medical Sciences and Peking Union Medical College, Beijing, China; 5 Department of Neuroscience and Regenerative Medicine, Medical College of Georgia at Augusta University, Augusta, GA 30912 USA

**Keywords:** Developmental biology, Neuroscience, Neurological disorders

## Abstract

Haplo-insufficiency of the *GATA3* gene causes hypoparathyroidism, sensorineural hearing loss, and renal disease (HDR) syndrome. Previous studies have shown that *Gata3* is required for the development of the prosensory domain and spiral ganglion neurons (SGNs) of the mouse cochlea during embryogenesis. However, its role in supporting cells (SCs) after cell fate specification is largely unknown. In this study, we used tamoxifen-inducible *Sox2*^*CreERT2*^ mice to delete *Gata3* in SCs of the neonatal mouse cochlea and showed that loss of *Gata3* resulted in the proliferation of SCs, including the inner pillar cells (IPCs), inner border cells (IBCs), and lateral greater epithelium ridge (GER). In addition, loss of *Gata3* resulted in the down-regulation of p27^kip1^, a cell cycle inhibitor, in the SCs of *Gata3-*CKO neonatal cochleae. Chromatin immunoprecipitation analysis revealed that GATA3 directly binds to *p27*^*kip1*^ promoter and could maintain the quiescent state of cochlear SCs by regulating *p27*^*kip1*^ expression. Furthermore, RNA-seq analysis revealed that loss of *Gata3* function resulted in the change in the expression of genes essential for the development and function of cochlear SCs, including *Tectb*, *Cyp26b1*, *Slitrk6*, *Ano1*, and *Aqp4*.

## Introduction

GATA-binding protein 3 (GATA3) belongs to the GATA family transcription factors that bind the DNA sequence GATA. Previous studies have shown that GATA3 plays a role in the development of many tissues, including T-cells^1–3^, parathyroid^4,5^, kidney^6–8^, and cochlea^9^. In humans, haplo-insufficiency of the *GATA3* gene causes hypoparathyroidism, sensorineural hearing loss, and renal disease (HDR) syndrome, also known as Barakat syndrome. Among these three phenotypes, all patients have some form of sensorineural deafness and many suffer from congenital deafness^10,11^, suggesting GATA3’s essential role in cochlear development.

The mammalian inner ear is derived from the otic placode that invaginates and then closes to form the otic vesicle. The subsequent morphogenesis of the roughly spherical otic vesicle gives rise to a complex structure containing three semicircular canals, two gravistatic receptors (saccule, utricle), and the cochlea in mammals^12,13^. The sensorineural organ of the cochlea (the organ of Corti) is composed of sensory hair cells (HCs) and surrounding supporting cells (SCs). During cochlear development, GATA3 is continuously expressed from otic placode to mature cochlea^14–16^. Studies have shown that disruption of *Gata3* results in abnormal cochlear sensory epithelium development and its inaccurate innervation^17,18^. Previously, we have used *Pax2-Cre* mice, which express Cre in the developing inner ear after E9.5^19^, to conditionally knock out *Gata3* and have shown that *Gata3* is required for the establishment of the prosensory domain and the survival of spiral ganglion neurons (SGNs)^15^. Recently, a study has shown that *Gata3* is crucial for the maturation of inner hair cells (IHCs)^20^. However, the role of *Gata3* in SCs after cell fate specification is largely unknown.

In this study, we used *Sox2*^*CreERT2*^ to conditionally knock out *Gata3* to investigate the role of *Gata3* in the SCs of the neonatal mouse cochleae. Our results revealed that the deletion of *Gata3* in neonatal cochleae led to the downregulation of p27^kip1^ expression and cell cycle re-entry of SCs. Furthermore, chromatin immunoprecipitation (ChIP) analysis showed that GATA3 directly binds to the promoter of *p27*^*kip1*^. Together, our data demonstrated that GATA3 maintains the quiescent state of SCs by regulating the expression of *p27*^*Kip1*^.

## Results

### Loss of *Gata3* in early postnatal cochlear SCs results in the hypertrophy of SCs and the expansion of GER

**Figure 1:**
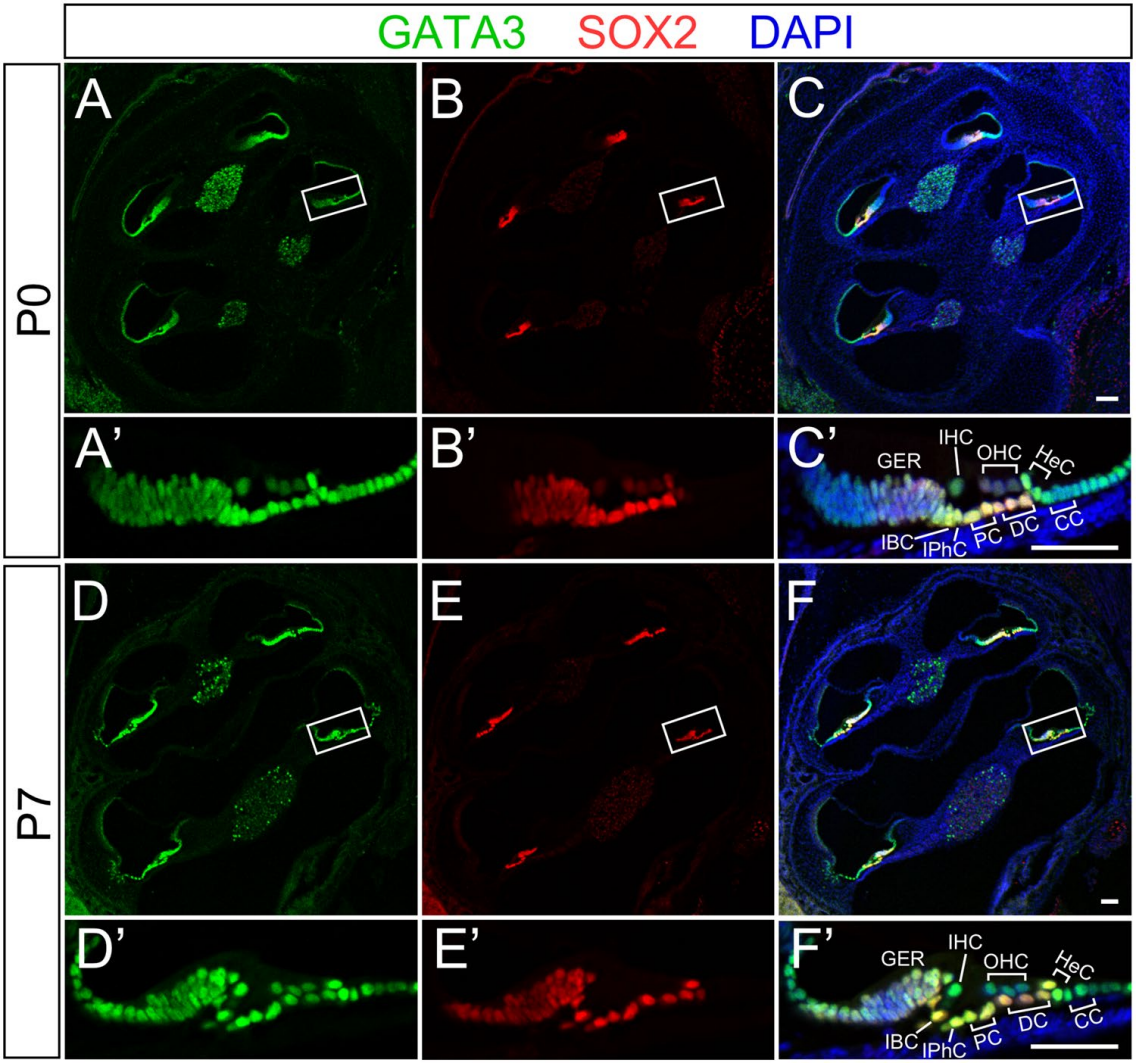
The expression of GATA3 in the neonatal cochlea. (**A**–**F**,**A**′–**F**′) Co-immunolabeling of GATA3 (green) and SOX2 (red) at P0 (**A**–**C**,**A**'–**C**′) and P7 (**D**–**F**,**D**′–**F**′) shows that GATA3 is expressed in all hair cells and SOX2^+^ supporting cells during neonatal cochlear development. *GER* greater epithelium ridge, *IBC* inner border cell, *IPhC* inner phalangeal cell, *PC* pillar cells, *DC* Deiters’ cells, *HeC* Hensen’s cells, *CC* Claudius’ cells, *IHC* inner hair cell, *OHC* outer hair cells. Scale bars equal to 100 μm in (**A**–**F**) and 50 μm in (**A**′–**F**′).

We first analyzed the spatiotemporal expression pattern of *Gata3* in the neonatal mouse cochlea. We co-labeled cochleae of wild type mice with antibodies against GATA3 and SOX2, a SC marker, and revealed that GATA3 was broadly expressed in the greater epithelial ridge (GER), inner border cells (IBCs), inner phalangeal cells (IPhCs), inner pillar cells (IPCs), outer pillar cells (OPCs), Deiters’ cells (DCs), Hensen’s cells (HeCs), HCs, Claudius’ cells (CCs), and cells in the outer sulcus at postnatal day 0 (P0) and P7 (Fig. 1A–F,A′–F′).

**Figure 2:**
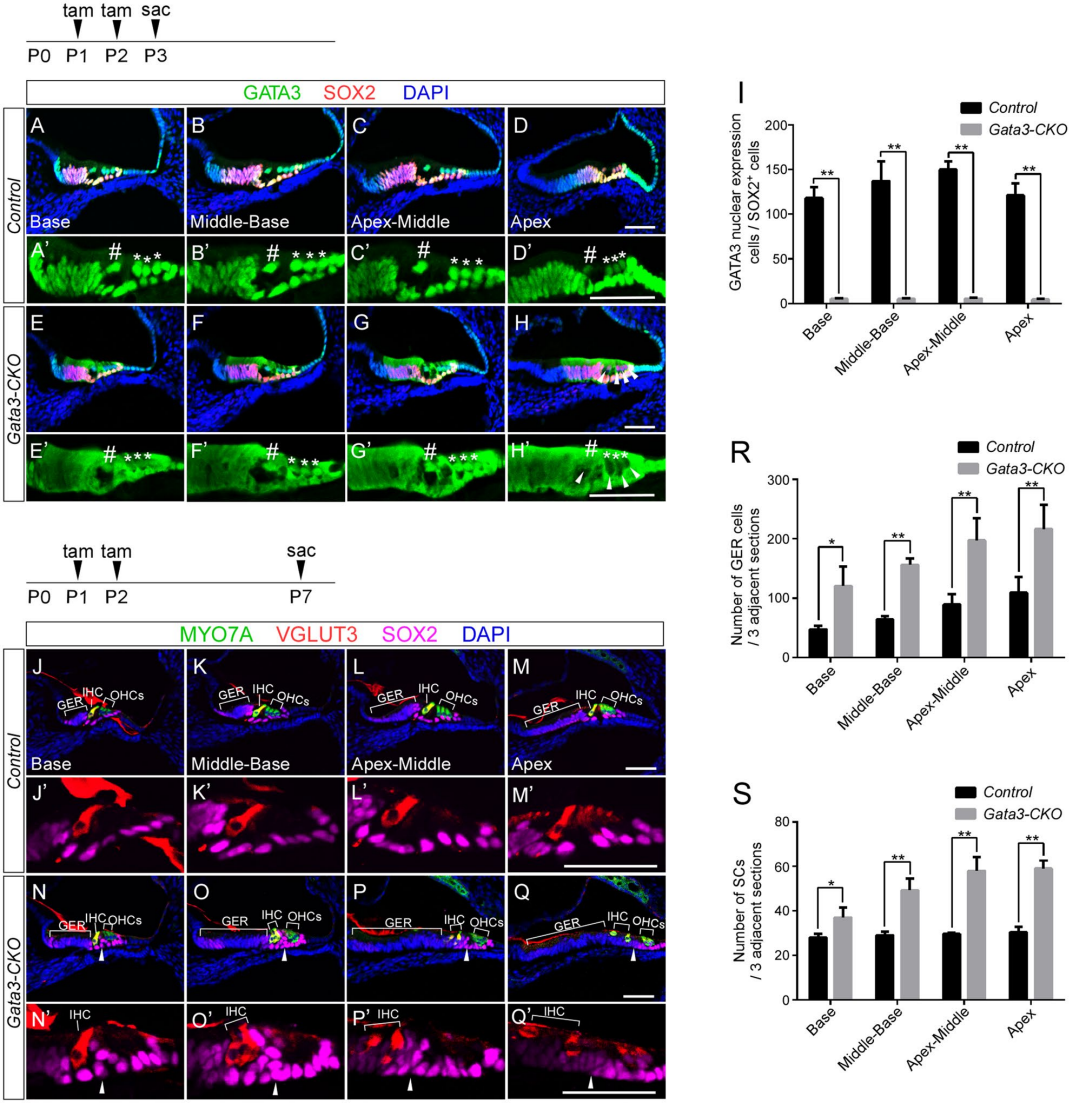
*Gata3* deficiency leads to increased number of SCs and expansion of the GER region. (**A**–**D**,**A**′–**D**′) The expression of GATA3 (green) is localized in the nuclei of SOX2^+^ (red) SCs in control cochleae at P3 after the administration of tamoxifen at P1 and P2. (**E**–**H**,**E**′–**H**′) In *Gata3-*CKO cochleae, the specific nuclear expression of GATA3 is lost in the SOX2^+^ SCs but the nuclear expression of GATA3 in HCs is not affected except at the apex (**H**,**H**′, arrowheads). IHCs and OHCs are indicated with pound sign (#) and asterisk (*), respectively. (**I**) Quantification of the number of SOX2^+^ SCs with GATA3 nuclear expression in 3 adjacent sections (a total thickness of ~ 54 μm) shows the high rate of *Gata3* deletion in *Gata3-*CKO mice. n = 3; **p < 0.01. (**J**–**M**,**J**′–**M**′) Immunolabeling of the control cochlea with anti-VGLUT3 (red), anti-MYO7A (green) and anti-SOX2 (magenta) reveals the normal pattern of SCs and the orderly arrangement of three rows of OHCs and one row of IHCs at P7 after the administration of tamoxifen at P1 and P2. (**N**–**Q**,**N**′–**Q**′) In *Gata3-*CKO cochleae, the orderly pattern of HCs is disrupted along with an increase in SCs (arrowheads) and an expansion of the GER region (bracket). (**R**,**S**) Quantification shows that the numbers of GER cells (**R**) and SCs (**S**) in 3 adjacent sections (a total thickness of ~ 54 μm) are significantly increased in *Gata3-*CKO cochleae compared with control mice. n = 3; *p < 0.05; **p < 0.01. Scale bars are 50 μm.

To investigate the role of *Gata3* in the SCs of the neonatal cochlea, we used the tamoxifen-inducible *Sox2*^*CreERT2*^ mice to conditionally delete *Gata3* in SCs. To generate tamoxifen-inducible, SC-specific *Gata3* conditional knockout mice (*Gata3*^*loxP/loxP*^*; Sox2*^*CreERT2/*+^), we first crossed *Gata3*^*loxP/loxP*^ mice with *Sox2*^*CreERT2/*+^ mice to generate *Gata3*^*loxP/*+^*; Sox2*^*CreERT2/*+^ mice. Then, crossing *Gata3*^*loxP/loxP*^ and *Gata3*^*loxP/*+^*; Sox2*^*CreERT2/*+^ mice produced *Gata3* conditional knockout mice. Without tamoxifen administration, *Gata3*^*loxP/loxP*^*; Sox2*^*CreERT2/*+^ mice were viable and phenotypically indistinguishable from control littermates. In tamoxifen-treated *Gata3*^*loxP/loxP*^*; Sox2*^*CreERT2/*+^ mice (*Gata3-*CKO mice hereafter), *Sox2*-CreERT2 is expected to remove the loxP-flanked exon 4 of *Gata3* in SCs, resulting in a reading frame-shift and a premature termination codon in exon 5, which deletes the two zinc finger domains and the rest of GATA3 C-terminal sequences essential for DNA-binding and nuclear localization^15^. We administered two tamoxifen injections to activate CreERT2 with a 24-h interval at P1 and P2, and assessed the efficiency of *Gata3* deletion at P3 by immunolabeling. The immunogen of the GATA3 antibody used in this study is encoded by exons 2 and 3. Thus, we expected to detect the truncated GATA3 without the zinc finger domains in the cytoplasm but not the nucleus in *Gata3-*CKO cochleae. Consistent with our prediction, the specific nuclear expression of GATA3, which was seen in the SCs of the control *Gata3*^*loxP/loxP*^ cochlea (Fig. 2A–D,A′–D′), was ablated in *Gata3-*CKO SCs (Fig. 2E–H,E′–H′), while the expression of GATA3 in HCs was not affected except at the apex (Fig. 2H,H′, arrowheads), likely due to active *Sox2* expression in apex HCs at P1 and P2. Quantification of GATA3 nuclear expression cells (SOX2^+^) demonstrates that two tamoxifen dosages at P1 and P2 are sufficient to inactivate *Gata3* in nearly all SOX2-expressing cells including cochlear SCs (Fig. 2I).

To analyze the effects of *Gata3* conditional knockout on neonatal cochlear SCs, we administered two tamoxifen injections to the mice at P1 and P2 and collected cochleae at P7. We used antibodies against SOX2, MYO7A, and VGLUT3 to identify SCs, HCs, and IHCs, respectively. The control mice exhibited a normal structure of the organ of Corti with three rows of OHCs and one row of IHCs surrounded by SCs arranged in order (Fig. 2J–M,J′–M′). In contrast, an increased number of SCs and an expansion in the GER region were observed in *Gata3-*CKO mice (Fig. 2N–Q,N′–Q′), as shown in quantification data (Fig. 2R,S). The increased SCs and GER expansion disrupted the organization of HCs, a single row of IHCs was inconsistently seen in the sagittal section (Fig. 2N–Q,N′–Q′).

### *Gata3* deficiency causes the abnormal proliferation of IPCs, IBCs, and lateral GER in the neonatal cochlea

**Figure 3:**
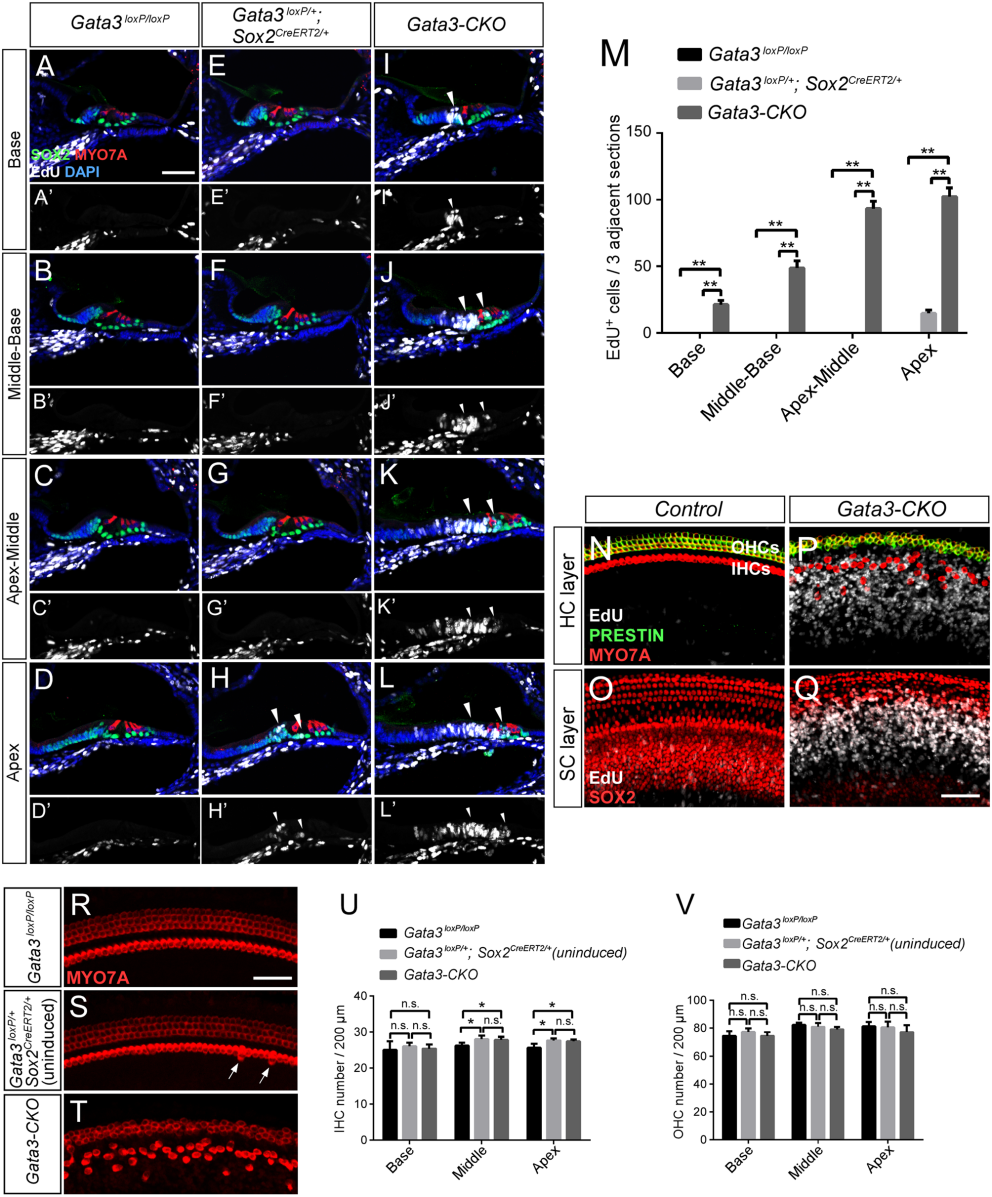
*Gata3* deficiency results in abnormal SC proliferation. (**A**–**D**,**A**′–**D**′) No EdU labeling (white) is detected in the SOX2^+^ SCs or GER region of *Gata3*^*loxP/loxP*^ cochleae at P7 after the administration of tamoxifen at P1 and P2, and daily EdU administration at P1–P6. (**E**–**H**,**E**′–**H**′) A small number of EdU^+^ cells are occasionally observed in the apex SCs or GER region of *Gata3*^*loxP/*+^*; Sox2*^*CreERT2/*+^ cochleae (**H**,**H**′, arrowheads). (**I**–**L**,**I**′–**L**′) In *Gata3-*CKO cochleae, numerous EdU^+^ cells are detected in PCs and the GER region (arrowheads). The number of EdU^+^ cells increases from the base to the apex of the cochlea. (**M**) Quantification of the EdU^+^ cells in 3 adjacent sections (a total thickness of ~ 54 μm) shows that the number of EdU^+^ cells is significantly increased in *Gata3-*CKO cochleae compared with *Gata3*^*loxP/*+^*; Sox2*^*CreERT2/*+^ cochleae. No EdU^+^ cell is detected in middle and basal turns of *Gata3*^*loxP/*+^*; Sox2*^*CreERT2/*+^ cochleae. The total number of proliferative cells gradually increases from base to apex in *Gata3-*CKO cochleae. n = 3; **p < 0.01. (**N**–**Q**) Whole-mount immunostaining shows the disorganization of HCs in *Gata3-*CKO cochleae as a result of SC proliferation. Antibodies against PRESTIN, MYO7A, and SOX2 are used to label OHCs, HCs, and SCs, respectively. No EdU^+^/MYO7A^+^ cell is observed in *Gata3-*CKO cochleae. (**R**–**T**) Whole-mount cochleae of *Gata3*^*loxP/loxP*^, *Gata3*^*loxP/*+^*; Sox2*^*CreERT2/*+^ (without tamoxifen), and *Gata3-*CKO mice are labeled with antibody against MYO7A. The middle turn of the cochlea is shown. Arrows indicate extra IHCs. (**U**,**V**) Quantification of IHCs (**U**) and OHCs (**V**) reveals the number of IHCs is slightly increased in apical and middle turns of *Gata3-*CKO cochleae compared with *Gata3*^*loxP/loxP*^ cochleae. However, there is no significant change in the number of HCs in *Gata3-*CKO cochleae compared with *Gata3*^*loxP/*+^*; Sox2*^*CreERT2/*+^ cochleae (without tamoxifen). n = 3 for *Gata3*^*loxP/*+^*; Sox2*^*CreERT2/*+^ cochleae and n = 5 for *Gata3*^*loxP/loxP*^ cochleae as well as *Gata3-*CKO cochleae; *p < 0.05; *n.s*. not significant. Scale bar 50 μm.

The observed increase in SCs and GER expansion in the neonatal cochleae of *Gata3-*CKO mice suggest that *Gata3* could play a role in maintaining SC quiescent state. To test this hypothesis, we treated mice with tamoxifen at P1 and P2, and with 5-ethynyl-2′-deoxyuridine (EdU) once a day from P1 to P6. Cochleae were harvested at P7 for the analysis of cell proliferation by Click-it EdU labeling. Consistent with previous studies that postnatal SCs are mitotically quiescent^21^, we observed no EdU^+^ cells in the SCs of the control *Gata3*^*loxP/loxP*^ cochleae (Fig. 3A–D,A′–D′). Previously, *Sox2* haplo-insufficiency has been shown to cause an increase in the number of inner HCs and the proliferation of inner pillar cells in the neonatal cochlea^22^. Thus, we investigated the change in cell proliferation in *Gata3*^*loxP/*+^*; Sox2*^*CreERT2/*+^ cochleae and detected a small number of EdU^+^ cells in the SCs and GER region of the apex turns (Fig. 3H,H′, arrowheads), but not in the basal and middle turns (Fig. 3E–G,E′–G′). In contrast, significantly more cells in GER and PC regions of *Gata3-*CKO mice were labeled with EdU throughout the length of the cochlear duct and the number of EdU^+^ cells increased from the base to the apex of the cochlea (Fig. 3I–L,I′–L′ arrowheads). Statistical analysis of the number of EdU^+^ cells confirmed that compared to the control *Gata3*^*loxP/loxP*^ and *Gata3*^*loxP/*+^*; Sox2*^*CreERT2/*+^ cochleae, the number of EdU^+^ cells was greatly increased in *Gata3-*CKO mice (Fig. 3M). Previous studies have shown that SC proliferation is accompanied by the generation of ectopic HCs^21,23–25^. However, no EdU^+^/MYO7A^+^ cells were observed in *Gata3-*CKO mice (Fig. 3I–L), indicating that no ectopic HC was generated from the proliferative SCs.

We then assessed whether the proliferative *Gata3-*CKO SCs gave rise to ectopic HCs using wholemount labeling of EdU and antibodies against PRESTIN, MYO7A, and SOX2. Compared to the quiescent state of HCs and SCs in the control mice (Fig. 3N,O), increased SC proliferation was detected in *Gata3-*CKO cochleae, which consequently displaced HCs (Fig. 3P,Q). Again, we observed no EdU^+^/MYO7A^+^ cells in *Gata3-*CKO mice (Fig. 3P). In addition, statistical analysis showed no significant change in the number of IHCs in the basal cochleae and of OHCs of the entire cochleae, the number of IHCs was slightly increased in the apical and middle turns of *Gata3*^*loxP/*+^*; Sox2*^*CreERT2/*+^ (without tamoxifen) and *Gata3-*CKO cochleae compared with those in *Gata3*^*loxP/loxP*^ cochleae (Fig. 3R–V, Table S4), suggesting that *Sox2* haplo-insufficiency could be the cause for the small increase of IHCs in apical and middle turns. The lack of significant difference in the number of IHCs and OHCs between *Gata3-*CKO mice and *Gata3*^*loxP/*+^*; Sox2*^*CreERT2/*+^ mice (without tamoxifen) also showed that the *Gata3* deficiency does not result in the generation of ectopic HCs. Taken together, these data indicate that *Gata3* deletion leads to cell cycle re-entry of a large number of SCs throughout the entire length of the cochlea but not cell fate conversion of SCs into HCs.

**Figure 4:**
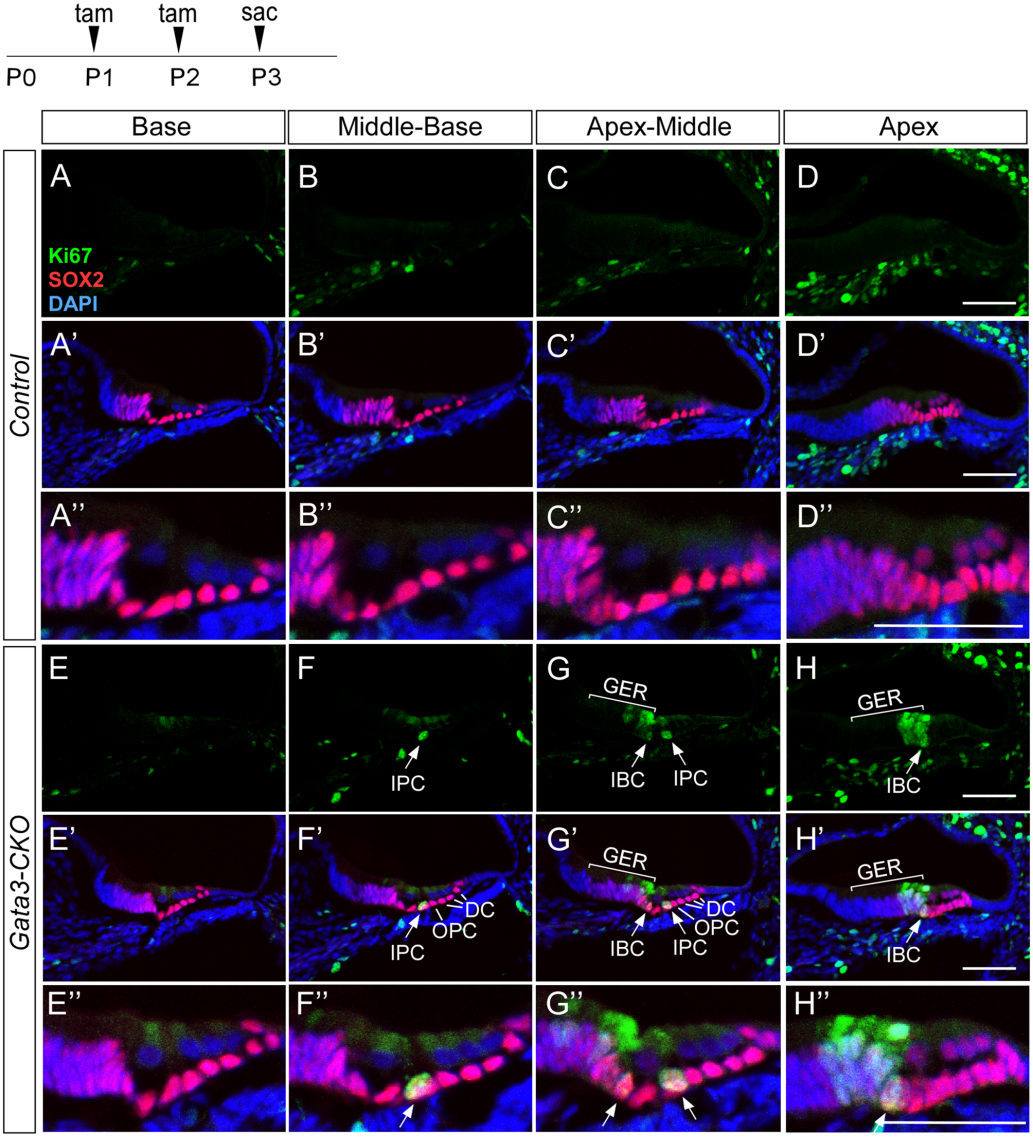
Deletion of *Gata3* results in the proliferation of IPCs, IBCs, and cells in the lateral GER. (**A**–**D**,**A**′–**D**′,**A**″–**D**″) Immunolabeling of Ki67 (green), a marker of cell proliferation, shows that SCs and GER have exited the cell cycle in control cochleae. (**E**–**H**,**E**′–**H**′,**E**″–**H**″) In *Gata3-*CKO cochleae, the expression of Ki67 is detected in IPCs, IBCs, and lateral GER. Scale bar 50 μm.

To determine the onset and origin of SC proliferation, we performed a time course analysis by tamoxifen administration at P1 and P2, and collecting cochlear sections at P3–P9 for immunolabeling with antibodies against SOX2 and Ki67, a proliferative marker. In contrast to the absence of Ki67 expression in the SOX2^+^ SCs of control mice (Fig. 4A–D,A′–D′,A″–D″), ectopic Ki67 expression was detected in IPCs, IBCs, and the lateral GER in *Gata3-*CKO mice at P3 (Fig. 4E–H,E′–H′,E″–H″). At P4-P5, there was an increasing number of Ki67^+^ proliferative cells restricted in these regions in *Gata3-*CKO mice compared to those of control mice (Fig. S1A,B). At P7, the Ki67 immunostaining signal began to wane (Fig. S1C) and mostly disappeared at P9, while the significant expansion of GER region remained (Fig. S1D). Thus, loss of *Gata3* causes the cell cycle re-entry of IPCs, IBCs, and the lateral GER, and the proliferation capability appears to be limited to these cells before P7. We also analyzed cochlear morphology at P12 to investigate the cell fate of extra SCs. We gave the mice tamoxifen injections at P1 and P2, and collected cochleae at P12. Compared with control mice, the *Gata3*-CKO mice did not have a typical inner sulcus (IS) structure but retained the expanded GER region (Fig. S2A–H).

### GATA3 maintains the quiescent state of cochlear SCs by directly regulating *p27*^*kip1*^ expression

**Figure 5:**
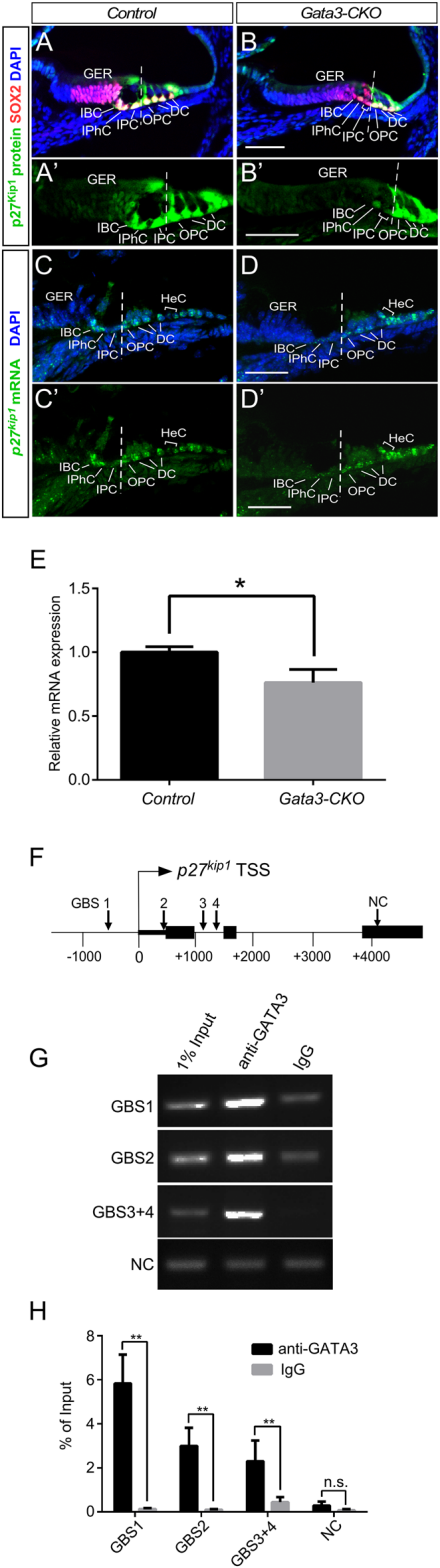
GATA3 binds to the *p27*^*kip1*^ promoter and is required for its expression in the cochlea. (**A**,**B**,**A**′,**B**′) *Gata3* deletion leads to the downregulation of p27^kip1^ expression (green) in IPCs, IBCs, and the lateral GER at P4 but not that of SOX2 (red). The middle turn of cochlea is shown. (**C**,**D**,**C**′,**D**′) RNAscope in situ hybridization shows the expression level of *p27*^*kip1*^ mRNA was decreased in *Gata3*-CKO mice cochleae at P4 after the administration of tamoxifen at P1 and P2. (**E**) RT-qPCR result shows that the expression of *p27*^*kip1*^ is significantly reduced in *Gata3-*CKO cochleae. n = 3; *p < 0.05. (**F**) Schematic drawing depicts the putative GATA3-binding sites (GBSs) of the *p27*^*kip1*^ promoter. (**G**) ChIP-PCR results show that anti-GATA3 immunoprecipitation specifically enriched the GBS-containing sequences. Control IgG and a coding region within exon 3 containing no GBS are used as negative controls for the ChIP-PCR analysis. (**H**) The results of ChIP-qPCR confirm that these predicted GBS-containing sequences are significantly enriched by ChIP with anti-GATA3. n = 4; *n.s*. not significant; **p < 0.01. Scale bar 50 μm.

Previous studies have revealed that the expression of p27^kip1^ a cyclin-dependent kinase inhibitor, is essential to maintain the quiescence of SCs and that the absence of *p27*^*kip1*^ leads to SC proliferation in neonatal cochlea^26–28^. In our previous study using *Pax2-*Cre to conditionally ablate *Gata3* in the developing inner ear, we have shown that *Gata3* is required for the establishment of the prosensory domain and that targeted deletion of *Gata3* abolishes p27^kip1^ expression in the presumptive prosensory region^15^. We thus investigated whether the observed cell proliferation in *Gata3-*CKO mice was caused by p27^kip1^ down-regulation. Cryosections of control and *Gata3-*CKO cochleae at P4 were immunolabeled for SOX2 and p27^kip1^. As shown in Fig. 5A,B,A′,B′, while p27^kip1^ expression was broadly detected in the SCs and GER region of the control cochlea, it was downregulated specifically in IPCs, IBCs, and the lateral GER of *Gata3-*CKO mice. Interestingly, these cells retained the expression of SOX2, which has previously been shown to directly modulate *p27*^*kip1*^ expression^26^. RNAscope in situ hybridization was also used to assess the change in *p27*^*Kip1*^ expression at mRNA level. The results showed the expression level of *p27*^*kip1*^ mRNA was decreased specifically in IPCs, IBCs, and the lateral GER while its expression in OPCs, DCs, and HeCs remains high in *Gata3*-CKO mice cochleae (Fig. 5C,D,C′,D′), consistent with the results of anti-p27^kip1^ immunolabeling. In addition, RT-qPCR analysis showed the expression of *p27*^*kip1*^ was significantly reduced in *Gata3-*CKO cochleae (Fig. 5E). Our data suggest that the abnormal proliferation of IPCs, IBCs, and lateral GER caused by *Gata3* deficiency could be due to the downregulation of p27^kip^ expression.

To determine whether GATA3 directly regulates *p27*^*kip1*^ expression, we first analyzed for the binding of GATA3 to *p27*^*kip1*^ promoter region in the publicly available GATA3 ChIP-seq databases^29–31^. GATA3 ChIP-seq data from different tissues or cell lines were downloaded from GEO (GSE20898, GSE92295, and GSE109109) and transferred from mm8/mm9 to mm10 using liftOver from UCSC^32^. All enriched ChIP-seq peaks (bed/bedGraph format) surrounding the *p27*^*kip1*^ gene were then uploaded to the UCSC genome browser. Four GATA3 motifs were identified in the promoter region of *p27*^*kip1*^ by using Homer^33^, FIMO^34^ and JASPAR^35^. These motifs are located in the enriched peaks and contain the predicted GATA3-binding sites (GBSs) (Fig. 5F, Fig. S3). To confirm these GATA3 motifs in the cochlea, we then immunoprecipitated the chromatin from wild type cochlear ducts at P2 using anti-GATA3 antibody and performed PCR to amplify the GBS-containing sequences. GATA3 binding to GBS1 and GBS2 could be specifically resolved by ChIP-PCR while the binding of GATA3 to GBS3 and GBS4 was assessed together as these two binding sites are tightly associated and could not be effectively separated by shearing (Fig. S4). ChIP-PCR (Fig. 5G, Fig. S5) and ChIP-qPCR (Fig. 5H) with anti-GATA3 antibody showed that these GBS-containing sequences were significantly enriched in the cochlear duct compared to IgG controls. Additionally, a coding sequence in exon 3 of *p27*^*kip1*^ was used as a negative control (NC) to show no significant enrichment with anti-GATA3 antibody. Thus, our results indicate that GATA3 directly binds to *p27*^*kip1*^ in vivo and could positively regulate its expression to maintain the quiescent state of cochlear SCs.

### Transcriptomic changes in the cochlear duct of *Gata3-*CKO mice

**Figure 6:**
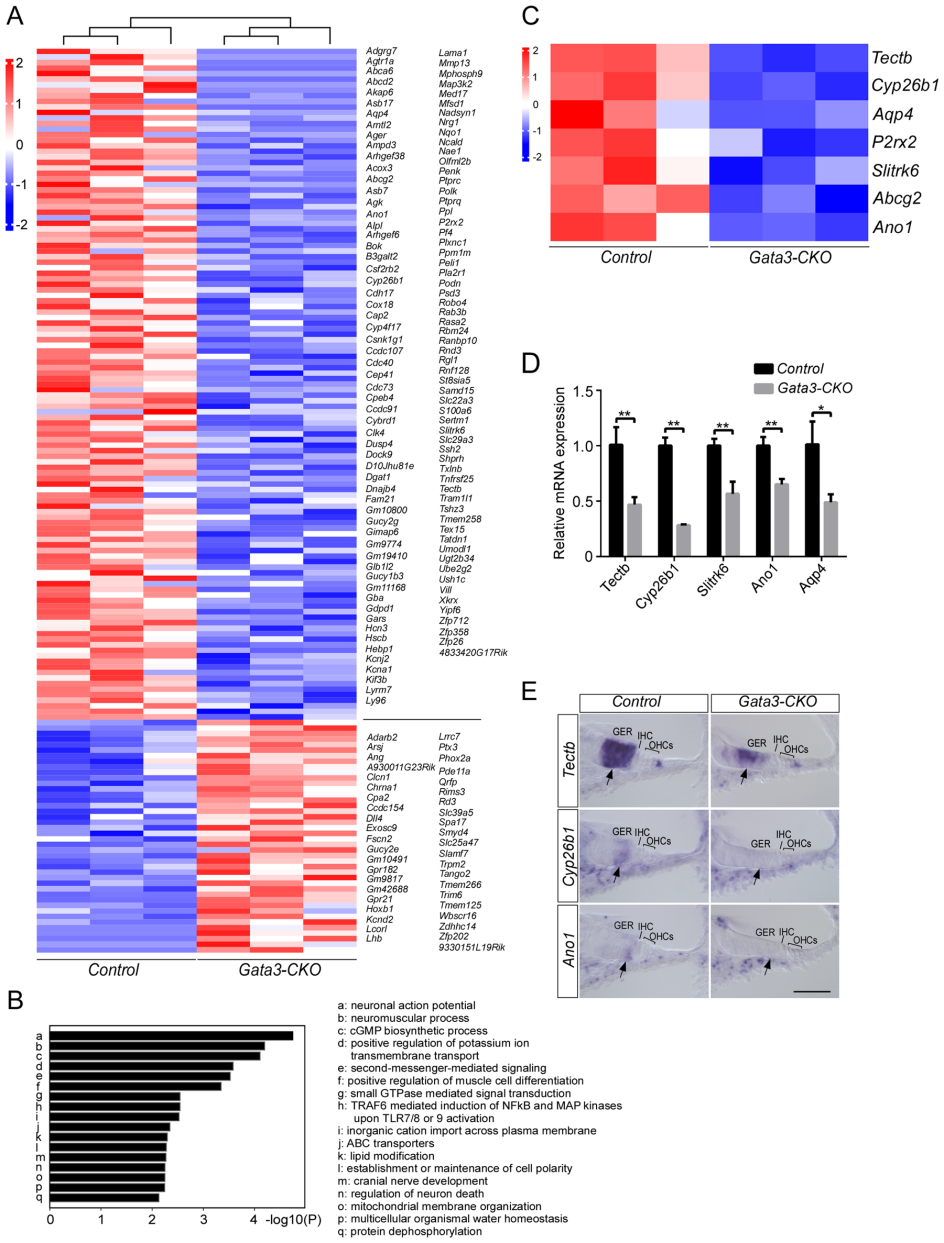
Transcriptome changes in the cochlear ducts of *Gata3-*CKO mice. (**A**) Heatmap represents the differentially expressed genes identified in the cochlear duct of *Gata3-*CKO mice at P4, and is divided into two clusters of control and *Gata3-*CKO mice. (**B**) The pathway and process enrichment analysis reveals multiple enriched terms, such as neuronal action potential, cGMP biosynthetic process, and positive regulation of potassium ion transmembrane transport. (**C**) Heatmap plot of the differentially expressed genes associated with SC function. (**D**) RT-qPCR validation of RNA-Seq results shows that the expression of *Tectb*, *Cyp26b1*, *Slitrk6*, *Ano1*, and *Aqp4* is significantly reduced in *Gata3-*CKO cochleae. n = 3; *p < 0.05; **p < 0.01. (**E**) ISH results confirm that the expression of *Tectb*, *Cyp26b1,* and *Ano1* is downregulated in the GER of *Gata3-*CKO mice. The middle turn of the cochlea is shown. Scale bar 50 μm.

To determine the changes in the transcriptome of *Gata3-*CKO cochlear ducts, we performed RNA-Seq analysis at P4 after tamoxifen administration at P1 and P2. To visualize transcriptomic differences between the control and *Gata3-*CKO cochleae, a heatmap was generated showing that the transcriptome pattern of cochlear duct was significantly changed in *Gata3-*CKO mice (Fig. 6A). We have identified a total of 163 differentially expressed genes (fold-change ≥ 2 and P ≤ 0.05) between control and *Gata3-*CKO cochleae. Among them, 121 genes were downregulated and 42 genes were upregulated in the *Gata3-*CKO cochleae (Table S5). Next, we categorized these highly differentially expressed genes between control and *Gata3-*CKO cochleae into enriched categories according to GO analysis, and revealed that multiple biological processes were disturbed in *Gata3-*CKO cochleae, including neuronal action potential, cGMP biosynthetic process, positive regulation of potassium ion transmembrane transport, second-messenger-mediated signaling, small GTPase mediated signal transduction, and inorganic cation import across plasma membrane. (Fig. 6B). Based on their relatively high sequencing depth (read count) and their known roles in SC function (Fig. 6C), we selected five significantly down-regulated genes in *Gata3-*CKO cochleae, *Tectb*, *Cyp26b1*, *Slitrk6*, *Ano1*, and *Aqp4*, for further analysis. As shown in Fig. 6D, the RNA-seq values of these five selected genes were verified by RT-qPCR. In addition, we performed in situ hybridization experiments to analyze the spatial expression of *Tectb*, *Cyp26b1*, and *Ano1*, and confirmed that consistent with the data obtained from the RNA-Seq analysis, the expression of *Tectb*, *Cyp26b1*, and *Ano1* were downregulated in the GER of *Gata3-*CKO mice (Fig. 6E arrow). Thus, disruption of *Gata3* alters the expression of genes involved in cochlear development and diseases.

## Discussion

*Gata3* deficiency causes human HDR syndrome. Previous studies have shown that *Gata3* is needed for the formation of the cochlear prosensory domain during embryonic development and for the survival and axonal projection of SGNs^15,17,18^. Moreover, a recent study has shown that *Gata3* is also involved in IHC maturation^20^. In the present study, we used an inducible *Sox2*^*CreERT2*^ line to delete *Gata3* in the neonatal mouse cochlea. Our results have revealed that *Gata3* is required to maintain the quiescent state of cochlear SCs and that the absence of *Gata3* in SCs leads to the proliferation of IPCs, IBCs, and the lateral GER. In this study, we have shown that *Sox2-*CreERT2-mediated deletion of *Gata3* is mostly limited to the SCs of the cochlea (Fig. 2). However, the expression of GATA3 in HCs was not affected except at the apex, which is likely due to active *Sox2* expression in HCs of the apex at P1 and P2. Our results are largely in agreement with the previous reporter expression study showing that *Sox2-*CreERT2 is expressed in ~ 20% HCs in the basal turns to ~ 84% in the apical turns with tamoxifen given at P0/P1 and in ~ 60% HCs in the apical turns with tamoxifen given at P1^36^. Since the expression of SOX2 is rapidly down-regulated in HCs between P0 and P2^37^, the small difference between our results and those by Walters et al.^36^ could be due to the slightly different timing of tamoxifen administration or due to the difference between the half-life of GFP and tdTomato reporter proteins and that of GATA3 or both.

During cochlear development, the terminal mitosis of sensory precursors occurs around E14.5, when the basal turn of the cochlea is the last region to exit the cell cycle^38–40^. Previous studies have shown that under certain experimental conditions, SCs of neonatal mouse cochlea also exhibit a limited proliferation capacity in vivo. For example, the disruption of cell cycle genes such as *p27*^*Kip1*^^26–28^ and *Rb*^41^ results in the cell cycle reentry of SCs. In addition, *Sox2*, which is critical to the development of the cochlea^42,43^, is required for the expression of p27^kip1^ in the developing cochlea and ablation of *Sox2* expression leads to the loss of p27^kip1^ expression in the prosensory domain at E14^44^. In neonatal mice, targeted deletion of *Sox2* results in the proliferation of IPCs and the absence of p27^kip1^ expression in IPCs, while other SCs remain quiescent. Conversely, *p27*^*kip1*^-null IPCs proliferate but retain SOX2 expression, consisting with the notion that SOX2 maintains the quiescent state of SCs by positively regulating p27^kip1^ expression^26^. Previously, we have shown that *Gata3* is required for the establishment of the prosensory domain. Targeted deletion of *Gata3* results in the ablation of p27^kip1^ expression and the significant downregulation of SOX2 expression in the developing cochlea, suggesting that *Gata3* should act upstream of *Sox2* and *p27*^*kip1*^^15^. In this study, we have observed abnormal proliferation and the downregulation of p27^kip1^ expression in the neonatal *Gata3-*CKO SCs, specifically in IPCs, IBCs, and the lateral GER. However, loss of *Gata3* function does not affect the expression of SOX2 in these cells. Our results indicate that while *Gata3* is essential for SOX2 expression during the establishment of prosensory domain in the embryonic cochlea, it is dispensable for maintaining SOX2 expression in neonatal SCs. Moreover, the decoupling of the expression of SOX2 and p27^kip1^ in *Gata3-*CKO SCs suggests that SOX2 might not act directly or not be sufficient by itself to regulate the expression of p27^kip1^. The reliance of p27^kip1^ expression on GATA3 also suggests that SOX2 could function in parallel with GATA3 to maintain p27^kip1^ expression in neonatal SCs.

Published studies have shown that SCs can spontaneously proliferate and form new HCs upon HC damage in the neonatal cochlea^21,23^. Downregulation of Notch signaling pathway and activation of Wnt signaling pathway also lead to the proliferation of SCs and the mitotic regeneration of HCs in the neonatal mouse cochlea^24,25,45^. In the present study, the observed SC proliferation in the neonatal cochleae of *Gata3-*CKO mice does not result in the de novo generation of ectopic HCs. The effect of *Gata3-*CKO mutation is consistent with those of *Sox2*-null or *p27*^*kip1*^-null mutations that cause SC proliferation but not a cell fate change because other factors such as ATOH1 are required in HC generation^26^. Our observation of the increased number of proliferating SCs from the base to the apex is also consistent with previous studies^25,45^. The gradual increase in SC proliferation from the base to the apex might be due to that cells in the apical turn are less mature. Previous studies have shown that the apical turn of the cochlea is the last region to acquire HC or SC fate during embryonic development^38,46,47^.

p27^kip1^, a member of the Cip/Kip family of cyclin-dependent kinase inhibitors, functions to prevent the activation of cyclin-CDK complexes and thus, controls cell cycle progression at G1 phase. In the cochlea, *p27*^*kip1*^ ablation stimulates the proliferation of SCs^26–28^. In this study, we have shown that p27^kip1^ expression is absent in the proliferating *Gata3-*CKO SCs, suggesting that *Gata3* could be an upstream regulator of *p27*^*kip1*^ to maintain the quiescent state of SCs. GATA3 ChIP-seq studies in other tissues have shown the presence of putative GBSs in the promoter region of *p27*^*kip1*^^29–31^. Our analysis of neonatal cochlear ducts has also demonstrated that these putative GBS-containing regions in the *p27*^*kip1*^ promoter are significantly enriched by anti-GATA3 antibody ChIP-PCR, arguing for the direct role of GATA3 in regulating *p27*^*kip1*^ expression to maintain the quiescent state of cochlear SCs. Nevertheless, we cannot rule out the possibility that GATA3 may also regulate *p27*^*kip1*^ in an indirect manner.

In addition, a recent study has shown that *Gata3* is negatively regulated by *p27*^*kip1*^ and that *p27*^*kip1*^ deletion results in the upregulation of GATA3 expression in SCs of mature cochleae. Ectopic GATA3 expression in conjunction with ATOH1 converts more SCs into HCs than ectopic ATOH1 expression alone, consistent with the result that combining *p27*^*kip1*^ deletion and ectopic ATOH1 expression results in an increased number of SCs converting to HCs in adult mice^48^. Collectively, these data and our results showing the dependence of p27^kip1^ expression on GATA3 indicate a mutual negative feedback relationship between GATA3 and p27^kip1^ as well as the essential role of GATA3 in HCs formation.

Due to the fact that *Gata3* is mainly removed from SCs, RNA-seq analysis on entire cochlear ducts fails to capture all relevant genes. Nevertheless, our RNA-seq analysis has identified a total of 163 genes that are differentially expressed in *Gata3-*CKO cochlear ducts. Among them are *Tectb*, *Cyp26b1*, *Slitrk6*, *Ano1*, and *Aqp4*, which have been implicated in cochlear development and diseases. *Tectb* encodes β-tectorin, a glycoprotein that is localized to the tectorial membrane (TM). Previous studies showed that *Tectb* knockout mice exhibit abnormal hearing with decreased sensitivity and sharper frequency selectivity caused by changes in TM wave properties^49,50^. CYP26B1, a member of the cytochrome P450 superfamily, is an enzyme involved in the metabolism of retinoic acid. Previous studies have reported that *Cyp26b1* is expressed in mesenchymal cells surrounding the otic epithelium at early embryonic stages. At later stages, *Cyp26b1* is confined to supporting cells in the cochlear and vestibular epithelia^51,52^. SLITRK6 is a member of the SLITRK family that consists of neuronal transmembrane proteins controlling neurite outgrowth. *Slitrk6* is strongly expressed in mouse cochlear SCs and *Slitrk6* deficiency results in a reduction in cochlear innervation as well as the loss of SGNs^53^. ANO1 is a Ca^2+^-activated Cl^-^ channel. It has been reported that the periodic excitation of IHCs in the prehearing cochlea is initiated by the buildup of K^+^ around IHCs due to ANO1 associated Cl^-^ efflux from nearby GER cells. The spontaneous and periodic activities of GER cells are mediated by the ATP/ purinergic autoreceptor/ Ca^2+^/ANO1 pathway^54^. AQP4 is a water channel that is involved in endolymph volume homeostasis and K^+^ homeostasis in the cochlea^55,56^. Taken together, these findings indicate that these genes may act as downstream genes of *Gata3* in cochlear SCs and that the altered expression levels of these genes may contribute to *Gata3* deficiency-induced sensorineural deafness in HDR syndrome.

Compared to a small reduction in *p27*^*kip1*^ expression detected by RT-qPCR (p = 0.020, Fig. 5E), our RNA-seq results revealed a small but insignificant reduction in the overall expression level of *p27*^*kip1*^ in *Gata3-*CKO mice compared with the control group (p = 0.358). In addition, no other factor involved in regulating cell cycle was identified by RNA-seq. p27^kip1^ is broadly expressed in the cochlear duct and *Gata3* deficiency leads to the downregulation of p27^kip1^ expression in only three cell types—IPCs, IBCs, and the lateral GER (Fig. 5). The failure to identify *p27*^*kip1*^ and other cell cycle-associated genes by RNA-seq could be due to an insufficient detection sensitivity of the RNA-seq approach on cochlear ducts. Future scRNA-seq approach could help confirm the cell type specific change in *p27*^*kip1*^ expression in *Gata3-*CKO SCs.

In summary, our study has revealed that *Gata3* deficiency induces the proliferation of IPCs, IBCs, and the lateral GER but no de novo HC generation in the neonatal mouse cochlea. In addition, the expression of p27^kip1^ is downregulated in *Gata3-*CKO mice compared with that in control mice. ChIP-PCR assays demonstrated that GATA3 directly binds to the promoter region of *p27*^*kip1*^. These data indicated that GATA3 regulates *p27*^*kip1*^ expression to maintain a quiescent state of IPC, IBC, and the lateral GER. Furthermore, GATA3 is required for the maturation and maintenance of SCs by regulating genes essential for SC function.

## Materials and methods

### Animals and treatment

*Sox2*^*CreERT2*^ (The Jackson Laboratory, Stock Number: 17593) and *Gata3*^*loxP*^ (previously generated in our lab) mouse strains are previously described^15,57^ and were maintained in C57BL/6J background. For Cre activation, tamoxifen (Sigma-Aldrich) in corn oil at a dosage of 40 μg/g body weight was administered into control and *Gata3-*CKO mice via intraperitoneal injection at P1 and P2. To perform the EdU assay, mice were intraperitoneally injected with EdU (75 mg/kg body weight, Invitrogen) once a day from P1 to P6. All animal procedures used in this study were approved by the University Committee of Animal Resources (UCAR) at the University of Rochester and Institutional Animal Care and Use Committee (IACUC) at Augusta University. All experiments were carried out in compliance with the ARRIVE guidelines, and all methods were carried out in accordance with relevant guidelines and regulations.

### Immunostaining and in situ hybridization

Tissue samples were fixed at 4 °C in 4% paraformaldehyde in phosphate buffered saline (PBS) for one hour to overnight according to the developmental stages. After fixation, samples were equilibrated in 30% sucrose in PBS prior to rapid freezing in OCT compound (TissueTek). Samples were then cryosectioned at 18 μm thickness. After permeabilization and blocking in PBS plus 0.2% Triton-X100 with 10% normal horse serum, sections were incubated with primary antibodies overnight at 4 °C. On the following day, sections were washed with PBS and incubated with secondary antibodies for 1.5 h at room temperature. The primary antibodies used in our experiments were rabbit anti-GATA3 (ab199428, Abcam, 1:500), rabbit anti-MYO7A (25-6790, Proteus Biosciences, 1:1000), goat anti-SOX2 (sc-17320, Santa Cruz, 1:500), guinea pig anti-VGLUT3 (AB5421-I, Millipore, 1:1000), goat anti-PRESTIN (sc-22692, Santa Cruz, 1:200), rabbit anti-Ki67 (VP-RM04, Vector Lab, 1:500), and rabbit anti-p27^kip1^ (ab32034, Abcam, 1:100). Alexa Fluor-conjugated donkey anti-guinea pig, anti-rabbit or anti-goat (Invitrogen, 1:1000) was used as the secondary antibody. Nuclei were stained with 4′,6-diamidino-2-phenylindole dihydrochloride (DAPI, 1:20,000, Life Technologies). To detect EdU-labeled proliferative cells, Click-iT EdU cell proliferation kit for imaging (Invitrogen) was used per manufacturer’s instruction.

For whole-mount immunostaining, whole inner ears were isolated under a dissecting microscope and were fixed in 4% paraformaldehyde at 4 °C overnight with a small hole in the apex. The inner ears were decalcified in 0.2 M EDTA in PBS for 1–5 days according to the developmental stages and cochleae were isolated under a dissecting microscope. After permeabilization and blocking in 0.1% PBST plus 0.03% saponin and 10% horse serum, cochleae were incubated with primary antibodies overnight at 4 °C. On the next day, cochleae were washed with PBS and were incubated with the fluorescently labeled secondary antibodies at 4 °C overnight. Sections and whole-mounts were analyzed using a Zeiss LSM 510 META confocal microscope.

For in situ hybridization (ISH), cryosections were prepared as described above. The primers listed in Table S1 were used to amplify the last exons of genes of interest for subsequent RNA-probe labeling. Digoxigenin-labeled RNA probes were transcribed by T7 RNA polymerase. ISH was performed as previously described^58^.

For RNAscope in situ hybridization, RNAscope Multiplex Fluorescent Reagent Kit v2 (Advanced Cell Diagnostics (ACD)) was used according to the manufacturer’s instructions.

### Chromatin immunoprecipitation (ChIP)

For ChIP analysis, cochlear ducts were dissected from wild type C57BL/6J mice at P2. ChIP was performed using the Magna ChIP A/G kit (Millipore) according to the manufacturer's instructions with minor modifications. Briefly, the cochlear ducts were crosslinked with 1% formaldehyde for 15 min, and the reaction was stopped by the addition of glycine. The samples were homogenized using a Dounce homogenizer. The nuclei were isolated using cell lysis buffer and resuspended in NEBuffer 3.1 (New England Biolabs Inc.). The samples were digested with 200 U of the restriction enzymes *FokI* and *StyI* (New England Biolabs Inc.) for 4 h at 37 °C and subsequently with 100 U of each enzyme for an additional 16 h at 37 °C. The nuclei were then incubated with an additional 200 U aliquot of each enzyme for 2 h at 37 °C^59^. Thereafter, the samples were spun down and resuspended in nuclear lysis buffer. A Covaris E220 sonicator (Covaris) was used to further shear and release chromatin (PIP: 105, duty factor: 2%, CPB: 200, treatment time: 8 min). The supernatant of the sonicated solution was used for immunoprecipitation with rabbit anti-GATA3 (ab199428, Abcam) via an established protocol. GATA3-binding site (GBS)-containing sequences were detected in the precipitated material by standard PCR and qPCR. The primers used are listed in Table S2. A coding sequence in exon 3 of *p27*^*kip1*^ was used as a negative control (NC).

### RNA-sequencing (RNA-seq)

Cochlear ducts at P4 were removed under a dissecting microscope. RNA extraction was carried out using Direct-zol RNA kit (Zymo Research) following the manufacturer’s protocol. The RNA-seq library was constructed using a KAPA Stranded mRNA-seq Kit (KK8420). Sequencing was performed on Novaseq (Illumina). The strand marked with dUTP was not amplified, allowing strand-specific sequencing. RNA-seq was performed in three biological replicates. The FASTX-Toolkit (http://hannonlab.cshl.edu/fastx_toolkit/index.html) was utilized for quality control of the RNA-seq data. Adaptors were first removed, and short reads were filtered out if they contained more than 10% ambiguous bases or more than 50% low-quality bases (Q < 5). Clean reads were mapped to the mouse genome (mm10) with TopHat (v2.1.0)^60^. The read counts for each gene were calculated with HTseq, and the differentially expressed genes were identified by using DESeq2 (cutoff fold-change ≥ 2 and P value ≤ 0.05)^61,62^. A heatmap was constructed using the heatmap.2 function of the R package gplots (v3.0.1.1). Pathway and process enrichment analyses were performed using Enrichr (http://amp.pharm.mssm.edu/Enrichr/).

### Reverse transcription-quantitative polymerase chain reaction (RT-qPCR)

The cochlear ducts of 4 mice at P4 were isolated under a dissecting microscope. Total RNA was extracted with the RNeasy Mini Kit (Qiagen) in accordance with the manufacturer’s protocol. An Iscript cDNA synthesis kit (Bio-Rad Laboratories) was used to generate cDNA libraries. qPCR was performed using SsoAdvanced Universal SYBR Green Supermix (Bio-Rad Laboratories) on CFX96 Real-Time PCR system (Bio-Rad Laboratories) according to the manufacturer's protocol. The housekeeping gene β-actin was used as the control. The primers used in these assays are listed in Table S3.

### Data analysis

Quantifications throughout the paper were performed blind to the genotype. All values are represented as the mean ± SEM and SPSS (v19.0) was used for data processing. Statistical analysis was performed using two-tailed Student’s t test or ANOVA with post hoc test. P values of less than 0.05 were considered significant. For all experiments, n values represent biological replicates. For quantification of GER cells, GER region was defined between the inner side of IHC and the modiolar edge of the cochlear duct.

## Supplementary Information

Supplementary Information.

## Data Availability

Data available from the authors upon request.
