# Supplementary material for: GATA3 maintains the quiescent state of cochlear supporting cells by regulating p27kip1
Source: Sci Rep. 2021 Aug 4;11:15779. doi: 10.1038/s41598-021-95427-3 (PMC8338922; doi:10.1038/s41598-021-95427-3)
Supplement: Supplementary file 1 — Supplementary Information. [file 41598_2021_95427_MOESM1_ESM.pdf]

SUPPLEMENTARY MATERIAL

Title: GATA3 maintains the quiescent state of cochlear supporting cells by regulating $p27^{kip1}$

Authors: Jiadong Xu[1,2], Dongliang Yu[3], Xuhui Dong[2,5], Xiaoling Xie[2,5], Mei Xu[1,2], Luming Guo[1,2], Liang Huang[5], Qi Tang[2,4], Lin Gan[2,5*]

Affiliations: [1]College of Life Sciences, Zhejiang University, Hangzhou, Zhejiang 310058, China;

[2]Department of Ophthalmology and Flaum Eye Institute, University of Rochester, Rochester, NY 14642;

[3]College of Life Sciences and Medicine, Zhejiang Sci-Tech University, Hangzhou, Zhejiang 310018, China;

[4]Department of Otolaryngology, Peking Union Medical College Hospital, Chinese Academy of Medical Sciences and Peking Union Medical College, Beijing, China;

[5]Department of Neuroscience and Regenerative Medicine, Medical College of Georgia at Augusta University, Augusta, GA 30912

***Correspondence:** Lin Gan, Department of Neuroscience and Regenerative Medicine, Medical College of Georgia at Augusta University, Augusta, GA 30912, Email: ligan@augusta.edu

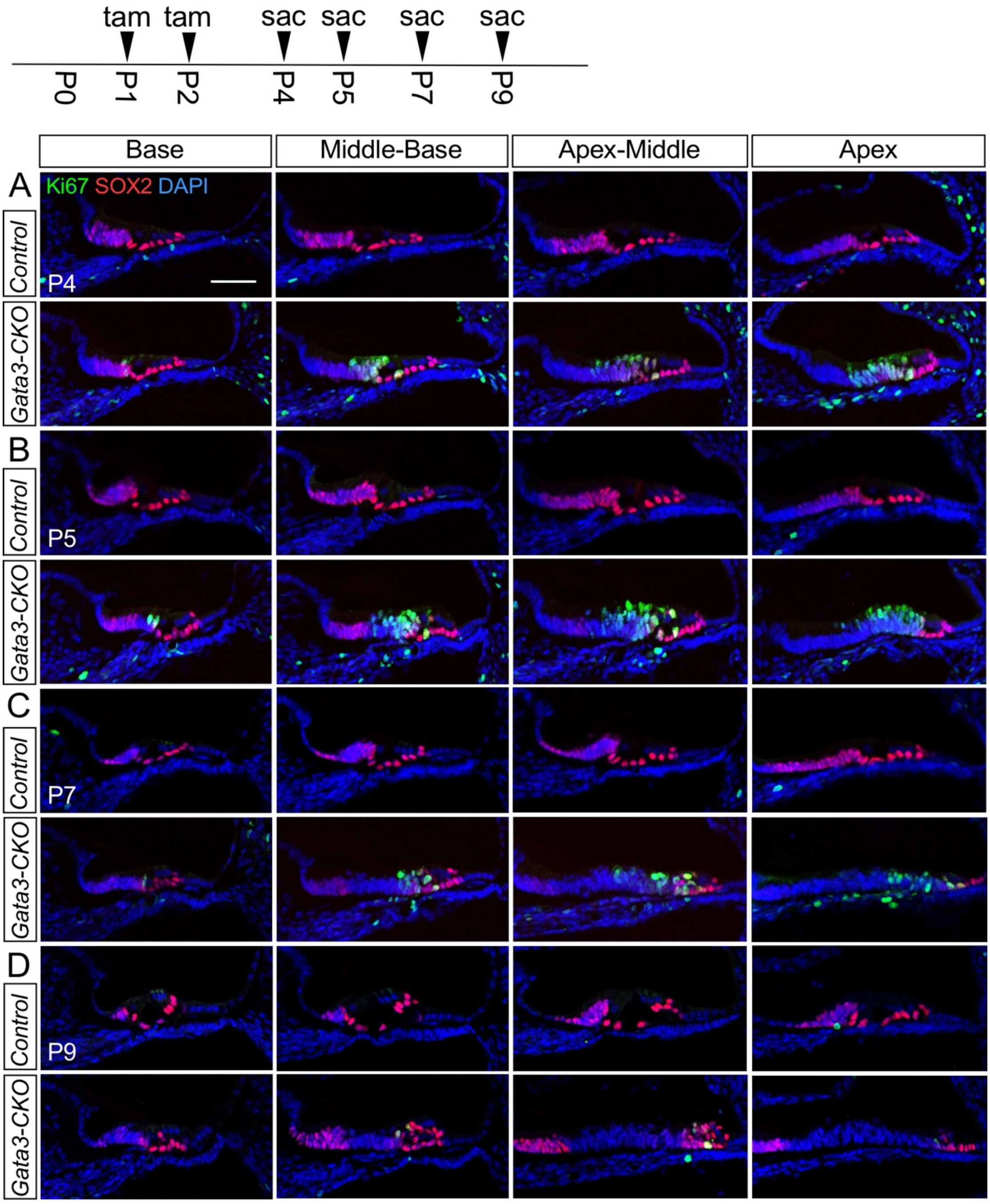

Figure S1. The proliferative capacity of *Gata3*-CKO SCs declines with age. Immunolabeling of Ki67 (green) shows an increasing number of Ki67^{+} proliferating SCs in *Gata3*-CKO mice at P4 (A) and P5 (B). The number of Ki67^{+} proliferating SCs decreases in *Gata3*-CKO mice at P7 (C) and only a small number of Ki67^{+} proliferating SCs are seen in *Gata3*-CKO mice at P9 (D). Scale bar, 50 μm.

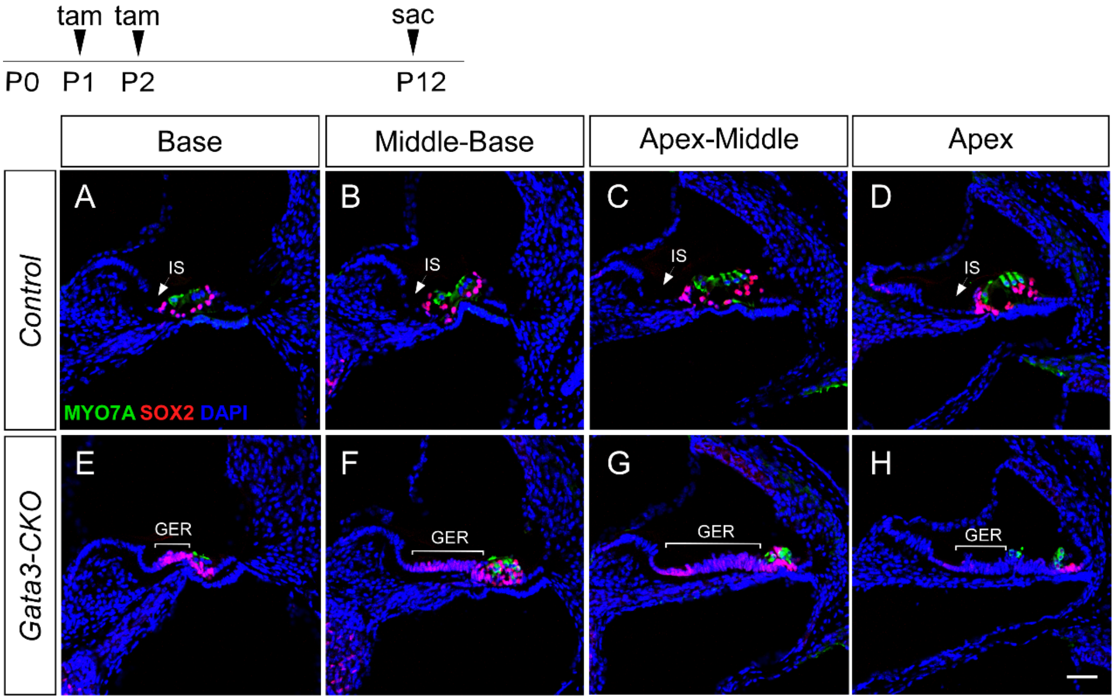

Figure S2. Extra SCs are retained in *Gata3*-CKO cochleae at later stage. (A-D) Control mice reveal the normal pattern of SCs and inner sulcus (IS) at P12 after the administration of tamoxifen at P1 and P2. (E-H) *Gata3*-CKO mice do not form typical IS structure but retain the expanded GER region and extra SCs. Scale bar, 50 μm.

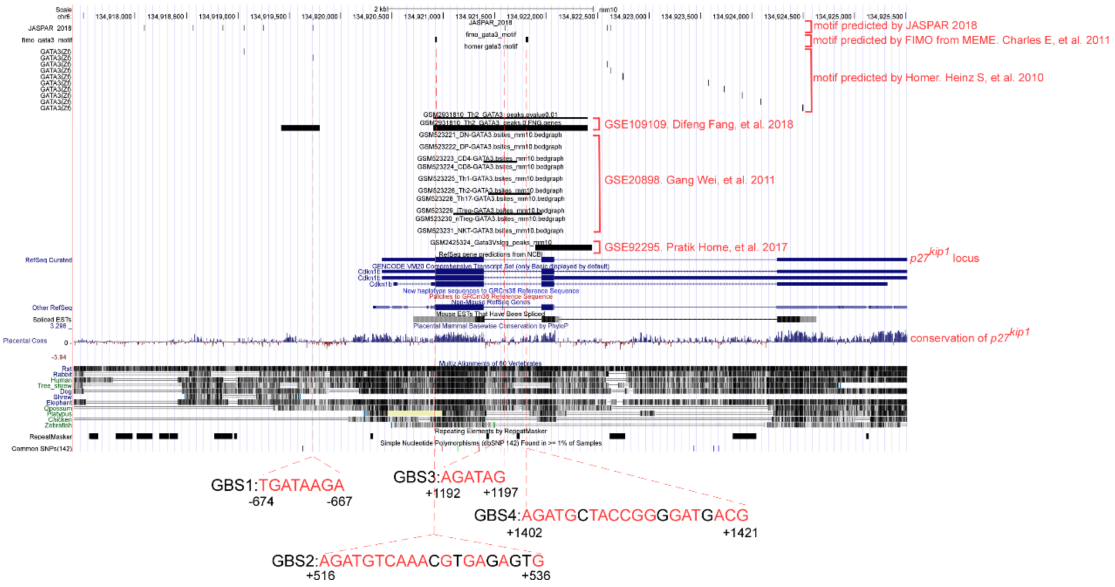

Figure S3. Summary of DNA motifs containing GATA3 binding sites at *p27*^{kip1} locus. Publicly available GATA3 ChIP-seq data from different tissues or cell lines were downloaded from GEO (GSE20898, GSE92295 and GSE109109). All ChIP-seq enriched peaks (bed/bedGraph format) were then uploaded to the UCSC Genome Browser. The GATA3 motifs surrounding *p27*^{kip1} were predicted by Homer, FIMO and JASPAR, which were also uploaded to UCSC genome browser. Motifs located in enriched peaks were considered as the predicted GATA3-binding sites (GBSs).

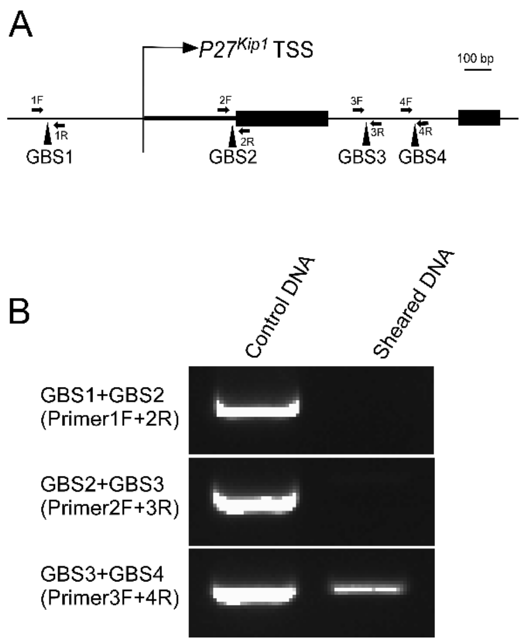

Figure S4. Examination of DNA shearing. (A) Schematic drawing showing the GBS positions and primer locations. (B) PCR analysis shows that GBS1 and GBS2 can be readily resolved using the described shearing method. However, the DNA fragment containing the closely linked GBS3 and GBS4 is not completely sheared apart. Thus, we analyze GBS3 and GBS4 together in this study.

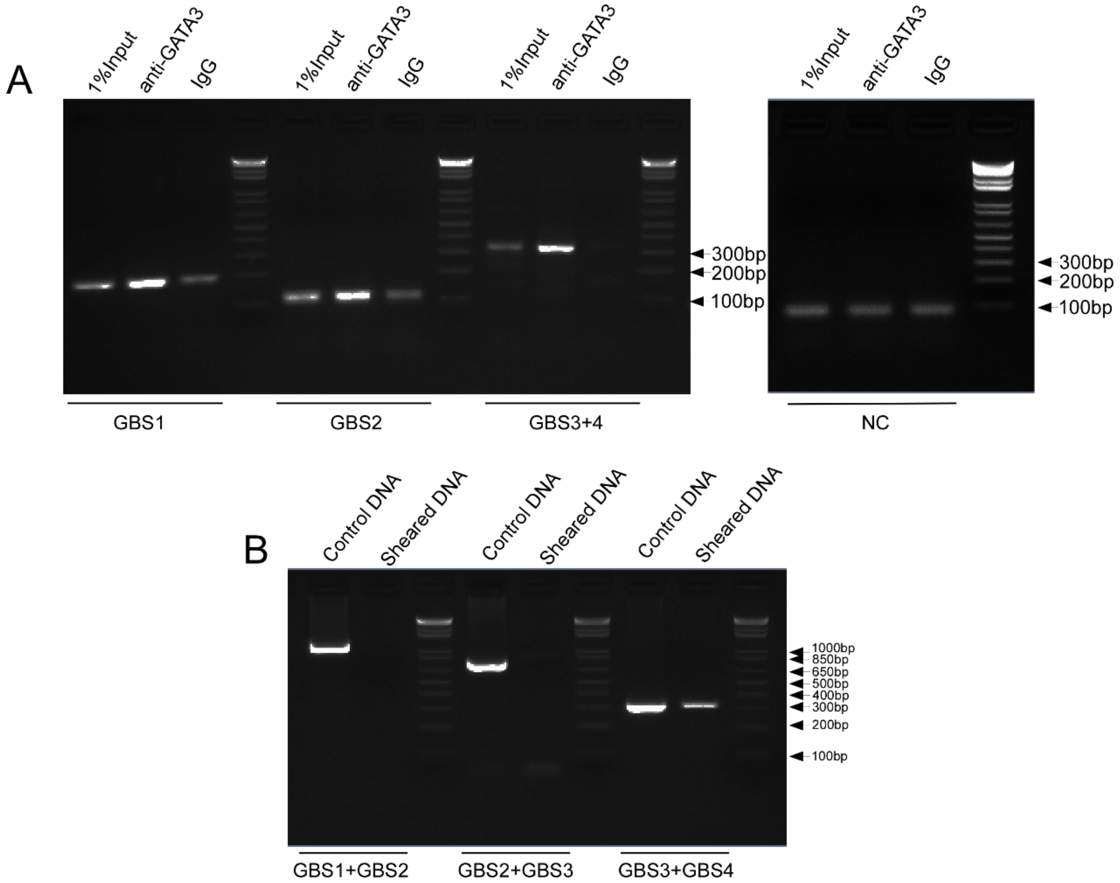

Figure S5. (A) Uncropped image of ChIP-PCR depicted in Fig. 5G. (B) Uncropped image showing the examination of DNA shearing depicted in Fig. S4.

Table S1. Primers used for in situ hybridization (T7 promoter is italicized)

Gene name	Forward primer	Reverse primer
Tectb	5’-GAGCCACACTTCCACTTATTCC-3’	*5’-TAATACGACTCACTATAGGGAGA*GTGCTGTCATGTGATGTCATTG-3’
Cyp26b1	5’-TCTGCCCCTTTGCTCTTG-3’	*5’-TAATACGACTCACTATAGGGAGA*ACAGGGATCCCCTTCAGC-3’
Ano1	5’-AGGGTGAGGCATGTTCCA-3’	*5’-TAATACGACTCACTATAGGGAGA*CACTGTCTGCCTTGCCCT-3’

Table S2. Primers used for ChIP-standard PCR/qPCR

Target	Forward primer	Reverse primer
GBS1	5’-CAATGGCTTTCACTGTGCTTAG-3’	5’-CAGGAGGTGGTTTCAAAAAGAA-3’
GBS2	5’-GTTTCGCTTTTGTTCGGTTTT-3’	5’-CCGTTAGACACTCTCACGTTTG-3’
GBS3+4	5’-GTTCTGCTTTAGCTCTGGGAAA-3’	5’-CGGTAGCATCTAAGGACACAAA-3’
NC	5’-TTGCATAAAGAGAAGCAACCTG-3’	5’-CCGAAAGGGACACTACATCTTT-3’

Table S3. Primers used for real-time PCR

Gene name	Forward primer	Reverse primer
$P27^{kip1}$	5’-GTGGACCAAATGCCTGACTC-3’	5’-GTTCTGTTGGCCCTTTTGTTT-3’
Tectb	5’-GTCAGGGCCTTCGTTTTGCT-3’	5’-CTCGGGGATTTTAGTGATGATGG-3’
Cyp26b	5’-TCATCGGAGAGACTGGTCACT-3’	5’-GGTGCTCACTAGCTGGTGTTC-3’
Slitrk6	5’-ATTTCATATCGCACCAGGAAGT-3’	5’-CATTGGTGACTGGGACTGTAGA-3’
Ano1	5’-CCCGTGCCAGTCACCTTTTT-3’	5’-TCATCTGCTTCCGTTTCCAGT-3’
Aqp4	5’-TCCGTCTTCTACATCATTGCAC-3’	5’-GTGAACACCAACTGGAAAGTGA-3’

Table S4. The number of HCs per 200 μm cochlear length

		Gata3$^{loxP/loxP}$ (n=5)	*Gata3*$^{loxP/+}$; *Sox2*$^{CreERT2/+}$ (without tamoxifen, n=3)	*Gata3*-CKO (n=5)
IHC	Base	25.00 ± 1.095$^{n.s.}$	26.00 ± 0.5774$^{n.s.}$	25.40 ± 0.5099
	Middle	26.20 ± 0.3742*	28.00 ± 0.5774$^{n.s.}$	27.80 ± 0.3742
	Apex	25.60 ± 0.5099*	27.67 ± 0.3333$^{n.s.}$	27.40 ± 0.2449
OHC	Base	74.60 ± 1.435$^{n.s.}$	77.33 ± 1.453$^{n.s.}$	74.60 ± 1.166
	Middle	82.40 ± 0.7483$^{n.s.}$	81.00 ± 1.528$^{n.s.}$	79.20 ± 0.7348
	Apex	81.40 ± 1.327$^{n.s.}$	80.67 ± 2.333$^{n.s.}$	77.20 ± 2.223

Quantification of IHCs and OHCs at P7 after the administration of tamoxifen at P1 and P2. *Gata3*$^{loxP/+}$; *Sox2*$^{CreERT2/+}$ mice were not treated with tamoxifen. *$p<0.05$; n.s., not significant vs. *Gata3*-CKO mice.

Table S5. Identification of differentially expressed genes from the cochleae

Gene	log2 Fold Change	P value	*Control*-1	*Control*-2	*Control*-3	*Gata3*-CKO-1	*Gata3*-CKO-2	*Gata3*-CKO-3
Robo4	-5.07	1.40E-03	10.05	4.14	3.9	0	0	0
Hcn3	-4.35	1.74E-02	0.91	7.24	2.93	0	0	0
Lyrm7	-4.31	1.61E-02	3.65	5.17	1.95	0	0	0
Umodl1	-4.29	1.71E-02	4.57	2.07	3.9	0	0	0
St8sia5	-4.26	2.24E-02	7.31	2.07	0.98	0	0	0
Adgrg7	-4.18	2.23E-02	2.74	4.14	2.93	0	0	0
Csf2rb2	-4.03	3.79E-02	0.91	2.07	5.86	0	0	0
Agtr1a	-4	3.82E-02	2.74	1.03	4.88	0	0	0
Penk	-3.98	1.14E-02	5.48	8.27	2.93	0	0.95	0
Xkrx	-3.93	5.39E-03	81.29	98.22	0.98	5.05	3.82	2.94
Ugt2b34	-3.86	4.86E-02	1.83	3.1	2.93	0	0	0
Abca6	-3.64	3.49E-02	10.05	1.03	1.95	1.01	0	0
Txlnb	-3.62	3.25E-02	0.91	7.24	4.88	0	0	0.98
Abcd2	-3.59	3.11E-02	4.57	6.2	1.95	1.01	0	0
Akap6	-3.4	1.88E-02	0.91	11.37	8.79	2.02	0	0
Asb17	-3.08	3.65E-02	7.31	8.27	0.98	0	0.95	0.98
Tnfrsf25	-3.05	3.21E-02	6.39	3.1	6.83	1.01	0	0.98
Samd15	-2.98	4.31E-02	7.31	4.14	3.9	0	1.91	0
Nadsyn1	-2.92	1.40E-02	6.39	9.3	6.83	2.02	0	0.98
Ptprc	-2.9	4.52E-02	3.65	6.2	4.88	1.01	0.95	0
Rab3b	-2.88	4.82E-02	6.39	4.14	3.9	1.01	0	0.98
Gm10800	-2.86	1.40E-02	39.27	87.88	7.81	7.07	10.5	0.98
Gucy2g	-2.63	2.61E-07	52.97	43.42	30.26	3.03	10.5	6.86
Kcnj2	-2.61	1.64E-02	9.13	4.14	10.74	2.02	0.95	0.98
Ly96	-2.6	7.08E-05	82.2	26.88	9.76	8.08	8.59	2.94
Tectb	-2.57	1.15E-39	1410.18	1437.1	1024.94	154.58	241.59	257.78
Slc22a3	-2.5	2.84E-02	10.96	7.24	3.9	1.01	2.86	0
Cyp26b1	-2.45	2.53E-07	44.75	50.66	33.19	10.1	5.73	7.84
Cdh17	-2.38	3.70E-02	6.39	6.2	7.81	0	0.95	2.94
Aqp4	-2.37	3.77E-02	10.05	7.24	2.93	1.01	1.91	0.98
Arntl2	-2.37	3.73E-04	6.39	51.69	33.19	4.04	8.59	4.9
Hscb	-2.37	4.18E-02	10.96	5.17	3.9	0	1.91	1.96
Gimap6	-2.31	1.32E-02	7.31	9.3	12.69	3.03	0.95	1.96
Polk	-2.26	1.56E-02	9.13	11.37	7.81	3.03	1.91	0.98
Lama1	-2.24	4.86E-02	5.48	6.2	6.83	1.01	1.91	0.98
Bok	-2.24	1.88E-02	13.7	7.24	6.83	3.03	0.95	1.96
Tram1l1	-2.23	4.43E-02	11.87	2.07	8.79	0	1.91	2.94
Cox18	-2.19	3.53E-02	6.39	10.34	5.86	3.03	0.95	0.98

Ptprq	-2.17	1.42E-03	23.75	23.78	9.76	5.05	4.77	2.94
Nrg1	-2.17	2.87E-02	5.48	11.37	9.76	4.04	1.91	0
Rasa2	-2.12	1.90E-02	13.7	9.3	6.83	1.01	1.91	3.92
Gm9774	-2.12	4.25E-02	7.31	7.24	6.83	1.01	0.95	2.94
Cap2	-2.11	4.97E-02	10.96	6.2	3.9	1.01	0.95	2.94
Rbm24	-2.06	6.15E-03	18.27	19.64	11.71	8.08	0	3.92
Tshz3	-2.05	1.38E-02	8.22	20.68	7.81	2.02	4.77	1.96
Gm19410	-2.04	4.64E-02	10.05	4.14	9.76	0	3.82	1.96
Glb1l2	-2.02	1.66E-02	20.09	9.3	9.76	1.01	7.64	0.98
Cyp4f17	-2	4.20E-02	7.31	10.34	5.86	1.01	1.91	2.94
S100a6	-1.96	8.35E-03	9.13	14.47	18.55	5.05	2.86	2.94
Ager	-1.95	1.36E-02	19.18	12.41	9.76	2.02	7.64	0.98
Kcna1	-1.95	2.37E-02	9.13	14.47	6.83	3.03	2.86	1.96
Ppl	-1.91	2.19E-02	8.22	10.34	14.64	3.03	4.77	0.98
P2rx2	-1.86	1.21E-02	16.44	17.58	8.79	2.02	2.86	6.86
Sertm1	-1.84	4.98E-02	5.48	12.41	6.83	2.02	1.91	2.94
Ampd3	-1.81	9.10E-03	16.44	15.51	12.69	3.03	3.82	5.88
Tmem258	-1.79	2.57E-02	14.61	9.3	13.67	8.08	1.91	0.98
Csnk1g1	-1.76	2.86E-03	25.57	22.75	14.64	7.07	5.73	5.88
Slitrk6	-1.74	2.99E-04	37.45	45.49	25.38	10.1	16.23	5.88
Nqo1	-1.73	2.72E-02	13.7	11.37	10.74	4.04	0.95	5.88
Zfp712	-1.67	4.97E-02	11.87	8.27	7.81	3.03	3.82	1.96
Pf4	-1.63	2.40E-02	18.27	12.41	8.79	4.04	3.82	4.9
4833420G17Rik	-1.6	3.43E-02	25.57	5.17	10.74	5.05	3.82	4.9
Olfml2b	-1.58	2.87E-02	10.05	13.44	14.64	3.03	5.73	3.92
Mmp13	-1.58	2.21E-02	11.87	13.44	18.55	3.03	3.82	7.84
Dusp4	-1.55	2.51E-02	12.79	15.51	14.64	2.02	7.64	4.9
Ccdc107	-1.54	NA	28.31	21.71	350.43	57.59	38.2	42.15
Ranbp10	-1.47	1.36E-02	19.18	26.88	13.67	5.05	5.73	10.78
Plxnc1	-1.46	3.40E-02	16.44	11.37	12.69	4.04	3.82	6.86
Ube2g2	-1.45	2.00E-02	19.18	10.34	21.47	6.06	5.73	6.86
Cdc40	-1.45	9.15E-03	32.88	29.98	14.64	10.1	14.32	3.92
Slc29a3	-1.45	3.12E-02	15.53	18.61	11.71	8.08	1.91	6.86
Yipf6	-1.44	1.18E-02	24.66	15.51	23.43	9.09	10.5	3.92
Arhgef38	-1.43	3.23E-02	16.44	22.75	8.79	9.09	5.73	2.94
Gucy1b3	-1.43	2.36E-02	16.44	14.47	16.59	5.05	4.77	7.84
Acox3	-1.42	4.52E-03	21.01	37.22	23.43	9.09	10.5	10.78
Mphosph9	-1.38	1.25E-02	25.57	19.64	18.55	7.07	11.46	5.88
Abcg2	-1.36	6.71E-03	26.49	22.75	26.36	12.12	7.64	9.8
Cep41	-1.36	4.49E-03	45.67	45.49	16.59	11.11	17.19	13.72
Vill	-1.33	2.52E-02	30.14	17.58	11.71	11.11	5.73	6.86
Asb7	-1.33	4.03E-02	11.87	25.85	11.71	6.06	9.55	3.92
Gm11168	-1.33	1.62E-02	45.67	52.73	7.81	12.12	11.46	18.62

Ncald	-1.29	3.77E-02	19.18	18.61	12.69	6.06	3.82	10.78
Agk	-1.29	2.21E-02	37.45	13.44	20.5	5.05	12.41	11.76
Tex15	-1.29	1.71E-03	35.62	46.52	40.02	17.18	21.96	10.78
Hebp1	-1.26	3.06E-02	20.09	25.85	15.62	15.15	5.73	4.9
B3galt2	-1.26	2.10E-02	22.83	21.71	16.59	10.1	7.64	7.84
Ano1	-1.25	1.23E-03	52.97	50.66	32.21	20.21	17.19	19.6
Ppm1m	-1.22	2.93E-02	24.66	16.54	20.5	5.05	7.64	13.72
Cdc73	-1.21	1.41E-06	112.34	145.78	122.99	50.52	64.93	49.01
Peli1	-1.2	2.33E-02	29.23	18.61	19.52	8.08	13.37	7.84
Alpl	-1.2	1.74E-04	69.41	76.51	75.16	27.28	23.87	45.09
Zfp358	-1.19	2.96E-02	26.49	16.54	21.47	6.06	14.32	7.84
Rnd3	-1.19	1.87E-02	32.88	26.88	22.45	9.09	20.05	6.86
Dock9	-1.18	5.84E-03	47.49	49.63	22.45	15.15	19.1	18.62
Pla2r1	-1.16	4.49E-02	17.35	40.32	15.62	17.18	3.82	11.76
Arhgef6	-1.15	2.32E-02	17.35	21.71	35.14	11.11	10.5	11.76
Podn	-1.14	1.97E-02	61.19	34.12	16.59	15.15	23.87	11.76
Rgl1	-1.14	2.47E-03	47.49	37.22	44.9	18.19	21.01	19.6
Cpeb4	-1.14	3.02E-02	28.31	17.58	25.38	16.17	10.5	5.88
Gba	-1.12	2.50E-02	31.97	24.81	17.57	13.13	11.46	9.8
Psd3	-1.11	2.75E-02	28.31	25.85	17.57	12.12	12.41	8.82
Zfp26	-1.1	3.93E-02	23.75	18.61	18.55	8.08	8.59	11.76
Nae1	-1.09	2.22E-02	29.23	24.81	23.43	14.14	13.37	8.82
Map3k2	-1.09	4.43E-02	15.53	19.64	23.43	9.09	9.55	8.82
Gdpd1	-1.09	4.73E-02	16.44	25.85	18.55	12.12	5.73	10.78
D10Jhu81e	-1.08	1.21E-02	40.19	35.15	24.4	17.18	13.37	16.66
Kif3b	-1.07	2.19E-02	32.88	37.22	20.5	13.13	20.05	9.8
Rnf128	-1.07	3.83E-02	24.66	18.61	20.5	9.09	10.5	10.78
Med17	-1.07	4.82E-02	14.61	23.78	21.47	11.11	8.59	8.82
Ccdc91	-1.05	5.70E-03	38.36	51.69	49.78	14.14	24.83	28.42
Fam21	-1.04	3.65E-03	69.41	58.93	42.95	17.18	33.42	32.35
Dgat1	-1.04	3.20E-02	30.14	24.81	19.52	10.1	12.41	13.72
Cybrd1	-1.04	1.41E-02	46.58	61	21.47	18.19	22.92	21.56
Mfsd1	-1.03	1.02E-02	31.05	48.59	35.14	17.18	17.19	21.56
Gars	-1.02	8.18E-03	42.93	43.42	35.14	15.15	23.87	20.58
Ssh2	-1.02	3.41E-02	25.57	22.75	23.43	12.12	12.41	10.78
Ush1c	-1.02	8.76E-03	47.49	48.59	33.19	26.27	22.92	14.7
Shprh	-1.01	4.26E-02	21.92	27.91	19.52	13.13	12.41	8.82
Dnajb4	-1.01	4.74E-02	26.49	17.58	27.33	16.17	6.68	12.74
Clk4	-1.01	2.36E-02	33.79	37.22	25.38	9.09	19.1	19.6
Tatdn1	-1	3.45E-02	26.49	25.85	24.4	12.12	9.55	16.66
Clcn1	1.01	2.17E-02	17.35	16.54	12.69	32.33	39.15	22.54
Chrna1	1.03	4.50E-02	10.96	10.34	10.74	16.17	21.01	28.42
Ptx3	1.04	9.53E-05	42.93	54.8	72.23	96.99	127.96	123.5
Exosc9	1.08	6.07E-08	145.22	177.83	255.75	408.17	404.87	409.71

Lcorl	1.11	6.09E-06	54.8	61	81.02	136.39	162.33	124.48
Gucy2e	1.11	1.43E-02	9.13	15.51	17.57	24.25	33.42	33.33
Lhb	1.12	2.94E-02	8.22	8.27	17.57	31.32	21.01	21.56
Gm10491	1.15	1.80E-02	10.05	7.24	28.31	36.37	28.65	36.27
9330151L19Rik	1.15	4.35E-02	7.31	6.2	11.71	23.24	18.14	14.7
Phox2a	1.17	3.78E-02	6.39	6.2	17.57	30.31	22.92	14.7
Slc39a5	1.17	2.26E-02	9.13	10.34	9.76	22.23	21.96	21.56
Trpm2	1.19	4.04E-02	8.22	5.17	9.76	18.19	19.1	15.68
Gpr182	1.19	1.15E-02	8.22	15.51	15.62	36.37	31.51	21.56
Gm9817	1.25	3.74E-02	5.48	6.2	9.76	19.2	19.1	12.74
Wbscr16	1.29	2.17E-03	11.87	13.44	21.47	38.39	33.42	42.15
Zdhhc14	1.29	2.94E-02	6.39	10.34	5.86	18.19	12.41	24.5
Rims3	1.35	4.52E-02	3.65	4.14	8.79	14.14	16.23	11.76
Fscn2	1.37	2.56E-02	3.65	12.41	5.86	15.15	16.23	24.5
Qrfp	1.58	1.17E-02	3.65	7.24	6.83	13.13	22.92	16.66
Gm42688	1.6	3.13E-02	4.57	6.2	1.95	18.19	11.46	8.82
Rd3	1.61	2.35E-02	5.48	1.03	7.81	11.11	16.23	16.66
Tango2	1.61	4.64E-02	7.31	3.1	0.98	11.11	17.19	6.86
Spa17	1.85	4.56E-02	1.83	2.07	2.93	10.1	7.64	6.86
Cpa2	1.89	4.80E-02	1.83	3.1	1.95	3.03	10.5	11.76
Smyd4	1.92	2.50E-02	2.74	3.1	1.95	12.12	8.59	8.82
Dll4	2.05	1.08E-02	1.83	3.1	3.9	17.18	10.5	8.82
Lrrc7	2.47	3.06E-02	0	1.03	2.93	9.09	3.82	8.82
Ccdc154	2.5	2.77E-03	3.65	0	3.9	22.23	9.55	11.76
Tmem266	2.55	1.12E-02	1.83	2.07	0.98	5.05	7.64	15.68
Tmem125	2.58	4.26E-02	0	2.07	0.98	8.08	6.68	2.94
Zfp202	2.6	4.00E-02	1.83	1.03	0	4.04	7.64	5.88
Adarb2	2.71	4.90E-08	5.48	7.24	6.83	40.41	53.47	33.33
Slc25a47	2.95	1.19E-02	0.91	1.03	0.98	9.09	7.64	5.88
Arsj	3.01	3.84E-02	0.91	1.03	0	7.07	6.68	1.96
Pde11a	3.05	4.58E-02	1.83	0	0	9.09	5.73	0.98
Ang	3.26	1.88E-02	0.91	1.03	0	7.07	7.64	3.92
Kcnd2	3.61	3.05E-02	0.91	0	0	4.04	1.91	6.86
Slamf7	3.88	4.97E-02	0	0	0	4.04	0.95	2.94
Gpr21	4.05	3.29E-02	0	0	0	5.05	1.91	1.96
A930011G23Rik	4.34	1.53E-02	0	0	0	4.04	1.91	4.9
Trim6	4.34	1.77E-02	0	0	0	7.07	2.86	0.98
Hoxb1	4.67	5.77E-03	0	0	0	5.05	6.68	1.96
